# Transmission Dynamics of the Recently-Identified BYD Virus Causing Duck Egg-Drop Syndrome

**DOI:** 10.1371/journal.pone.0035161

**Published:** 2012-04-18

**Authors:** Naveen K. Vaidya, Feng-bin Wang, Xingfu Zou, Lindi M. Wahl

**Affiliations:** Department of Applied Mathematics, University of Western Ontario, London, Ontario, Canada; Albert Einstein College of Medicine, United States of America

## Abstract

Baiyangdian (BYD) virus is a recently-identified mosquito-borne flavivirus that causes severe disease in ducks, with extremely rapid transmission, up to 15% mortality within 10 days and 90% reduction in egg production on duck farms within 5 days of infection. Because of the zoonotic nature of flaviviruses, the characterization of BYD virus and its epidemiology are important public health concerns. Here, we develop a mathematical model for the transmission dynamics of this novel virus. We validate the model against BYD outbreak data collected from duck farms in Southeast China, as well as experimental data obtained from an animal facility. Based on our model, the basic reproductive number of BYD virus is high (*R*_0_ = 21) indicating that this virus is highly transmissible, consistent with the dramatic epidemiology observed in BYDV-affected duck farms. Our results indicate that younger ducks are more vulnerable to BYD disease and that ducks infected with BYD virus reduce egg production (to about 33% on average) for about 3 days post-infection; after 3 days infected ducks are no longer able to produce eggs. Using our model, we predict that control measures which reduce contact between mosquitoes and ducks such as mosquito nets are more effective than insecticides.

## Introduction

Sudden outbreaks of viral infection in ducks in April 2010 drew international attention [1] because those outbreaks were of unknown etiology, affected a huge population of ducks (over 4 million ducks in Fujian, Shandong and Zhejiang provinces of Southeast China alone), and caused dramatic losses in duck egg production resulting in serious economic loss in commercial farms; egg production was reduced within a flock by as much as 90% within 5 days of infection [2]. After the first outbreaks of egg-drop in some farms of Southeast China, the disease quickly spread to most of the duck-producing regions of China. A recent study by Su et al. [2] has concluded that the outbreaks were caused by a new flavivirus named Baiyangdian (BYD), and their findings have raised serious concern that due to the zoonotic nature of flaviviruses BYD may also pose a threat to human health [1], [3]–[5].

According to the systematic investigation by Su et al. [2], BYD is an RNA virus closely related to Tembusu and Sitiawan viruses (vector-borne flaviviruses). Given the devastating impact of BYD on duck farming, and the impending possibility of cross-species transmission, efficient mechanisms for the control of this virus are needed. Here, we use a carefully validated mathematical model, calibrated to available data, to gain an epidemiological understanding of the transmission dynamics of BYD, and to assess and compare control strategies.

We develop a transmission dynamic model and validate it using egg-production data collected from five infected flocks in Southeast China, as well as experimental data from an animal facility. We discuss the disease dynamics which characterize this novel virus and estimate the reproductive number. Our results reveal a large reproductive number (*R*_0_ = 21), consistent with field observations of extremely rapid disease transmission among ducks. We predict that once ducks are infected by BYD virus, their egg-producing capacity is reduced by more than 30% for about 3 days post-infection. Based on our model, after this 3-day early infection period, infected ducks completely lose their ability to produce eggs, consistent with experimental results that show severe hemorrhage of ovarian follicles 3 days post-infection. We further evaluate the effects of potential control strategies such as mosquito insecticides and mosquito-nets, and predict that control measures which reduce contact between ducks and vector organisms will be more effective than insecticides in maintaining egg production in infected flocks.

## Methods

### Mathematical model

Thorough genomic sequence analyses strongly support the hypothesis that BYD, like Tembusu and Sitiawan viruses, is primarily transmitted by mosquitoes [1], [2]. Here we adopt the suggestion by Su et al. [2] that BYD virus is transmitted among ducks by a vector organism, and draw upon the well-developed literature describing mosquito-borne malaria transmission [6]–[11] in developing our model. Although, based on the experimental evidence available to date, the transmission of BYD is most likely via vectors, we cannot exclude the possibility of direct transmission. We also investigated a model assuming direct transmission and found that the model including vector-borne transmission provided significantly better fits to the data (see discussion and supplementary materials).

A decline in egg-production is one of the key symptoms of ducks infected by BYD virus [2]. Moreover, histopathology of the clinical ovarian samples of ducks infected by BYD virus [2] shows mild hemorrhage of ovarian follicles in the early stage of infection, followed by severe hemorrhage in the late stage of the disease. We therefore divide the duck population into four mutually exclusive compartments: susceptible ducks (*S_d_*), early-stage infected ducks (*I_d1_*), late-stage infected ducks (*I_d2_*) and recovered ducks after infection by BYD virus (R*_d_*). The *I_d1_* class consists of ducks in the early stage of infection which have a reduced ability to produce eggs, while the ducks in the *I_d2_* class have negligible egg-producing capacity. We also consider susceptible mosquitoes (*S_m_*) and infected mosquitoes (*I_m_*).

**Figure 1:**
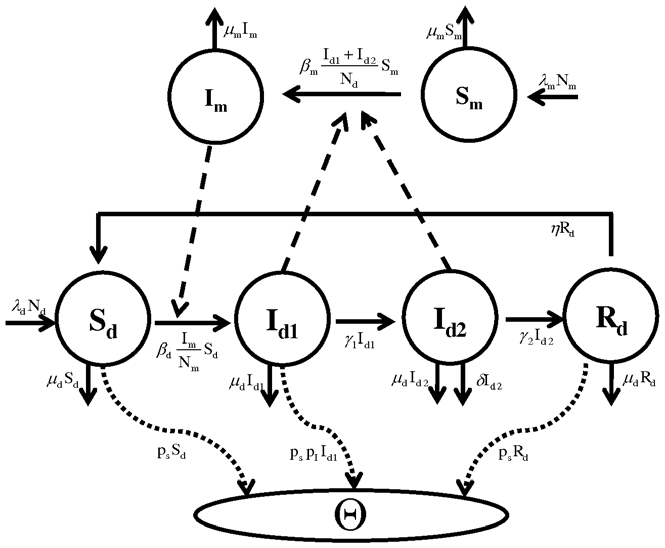
Schematic diagram of the model for transmission dynamics of BYD virus among ducks. *S_d_*: susceptible ducks, *I_d1_*: early-stage infected ducks, *I_d2_*: late-stage infected ducks, *R_d_*: recovered ducks, *S_m_*: susceptible mosquitoes, *I_m_*: infected mosquitoes, and 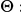 total egg production. Arrows indicate disease transmission, disease progression and egg production.

A schematic diagram of disease transmission and progression is presented in Fig. 1. While the basic principle of the model developed here is similar to the existing malaria models [8], our model differs in that infected ducks are classified into two groups (*I_1_* and *I_2_*) based on egg production capacity, and both classes, *I_1_* and *I_2_*, are infectious to susceptible mosquitoes. The mathematical model we consider is as follows:
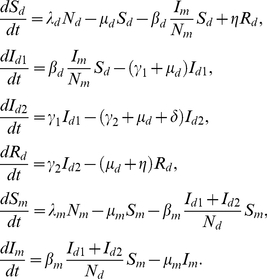
(1)Here, *N_d_ = S_d_+I_d1_+I_d2_+R_d_*, and *N_m_ = S_m_+I_m_* represent the total population sizes of ducks and mosquitoes, respectively. We assume that new ducks and new mosquitoes are recruited into the system proportional to their corresponding population sizes at rates *λ_d_* and *λ_m_*, respectively; *μ_d_* and *μ_m_* represent the natural death rates of ducks and mosquitoes, respectively. The parameter *γ_1_* is the rate of progression of infected ducks from the early to late stage, while *γ_2_* represents the recovery rate. The death rate due to BYD disease is represented by *δ* and the rate of immunity loss is represented by *η*.

In this model, new infected ducks are generated by mosquito-bites at a rate proportional to susceptible ducks, *S_d_*, and the probability that the mosquito is infectious, *I_m_*/*N_m_*, at an effective biting rate, *β_d_*. Thus, *β_d_* represents the product of the number of mosquito bites that one duck receives per unit time and the probability of successful transmission of BYDV from the mosquito to the duck, per mosquito bite. Similarly, new infected mosquitoes are generated at an effective biting rate, *β_m_*, when susceptible mosquitoes, *S_m_*, bite infected ducks, where the probability that the duck is infected is given by (*I_d1_+I_d2_*)/*N_d_*. The effective biting rate *β_m_* is the product of the rate at which one mosquito bites ducks, and the probability of successful transmission of BYDV from the duck to the mosquito, per mosquito bite.

In reality, the transmission rates *β_d_* and *β_m_* could be quite complex time-varying parameters (see Chitnis et al. [8]), which depend on the mosquito-duck ratio and other variables such as the mosquito's gonotrophic cycle (the amount of time a mosquito requires to produce eggs). Based on a field survey of larval habitats and mosquito densities [12] however, we observe that the mosquito-duck ratio remains nearly constant during the period of rapid BYD dynamics captured in the available data (up to 10 days). We therefore adopt a simplified approach, assuming *β_d_* and *β_m_* to be constant; we estimate these rates by fitting our model to available data.

The total egg production function is given by
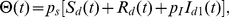
(2)where *p_s_* is the egg-production rate for susceptible or recovered ducks, and *p_I_* is the reduction in egg-production for early-stage infected ducks.

To simplify the problem, we introduce scaled variables 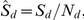 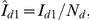 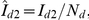 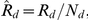 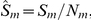 and 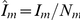. In this case, 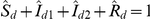 and 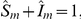 and thus we may consider only four equations:
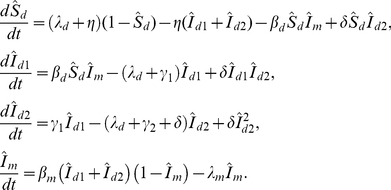
(3)Note that variables in Eq. (3) and hereafter represent the proportions in each compartment, not the actual number of ducks or mosquitoes. In the scaled variables, the egg production rate, 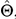 is given by
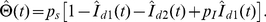
(4)


### Data and model validation

#### Egg production data

We obtained daily egg production data from two infected duck farms (Farm 1 and Farm 2) in Southeast China (Fig. 2 in Su et al. [2]). There are five sets of farm data, one from Farm 1 with a 35 week-old flock and 4 from Farm 2 (2-F1, 2-F2, 2-F3, 2-F4) with four different 76 week-old flocks. These data sets include daily egg production for 10 days. In addition, we used experimental egg production data for ducks infected in an animal facility [2], as described below.

**Figure 2:**
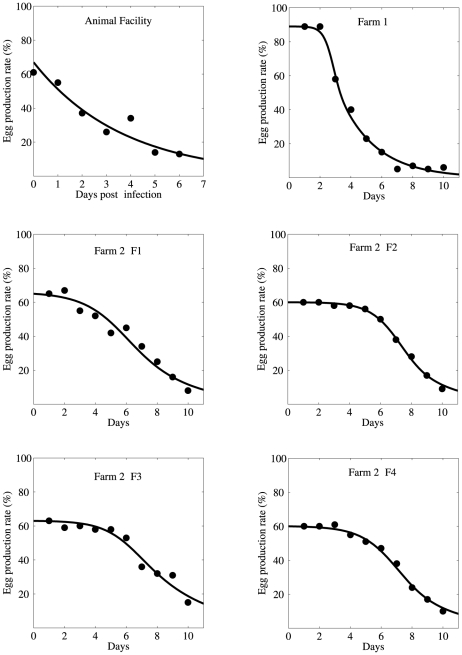
Egg production rate predicted by the model (solid line) along with the data (filled circles). The first figure shows experimental data from an animal facility and the remaining figures show natural outbreak data for duck flocks from two farms in southeast China. Egg production rate as expressed in % (y-axis) represents the percentage of ducks that produce eggs out of the total ducks in the flock on day *n*.

#### Model-fit to experimental data

In the experimental work described by Su et al. [2], after allowing ducks to adapt to the animal facility for 5 days to minimize the effect of shipping stress, 46 ducks were simultaneously infected by intramuscular and intranasal injection with BYD virus. Assuming all ducks exposed in this way become infected, we have 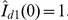 In this experimental setting there is no further susceptible ducks for new infection; 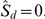 Moreover, during the observation period of about a week, no disease death was reported giving *δ* = 0, and no new duck was recruited giving 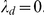 Therefore, the model for the dynamics of egg-producing ducks, the 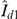 group, in the animal facility is reduced to
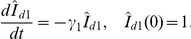
Solving, we find 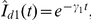 which gives the following formula for the egg production rate:
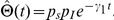
We perform a simple linear regression in MATLAB (MathWorks, Inc.) to fit the model to the data and estimate *γ_1_* and the product *p_s_p_I_*. Egg production by infected ducks in the animal facility as predicted by the model, along with the data, is shown in Fig. 2. Parameter estimates are provided in Table 1. Note that in reporting *p_I_* we have assumed that maximally favorable conditions were maintained within the animal facility, that is, *p_s_* = 1.

**Table 1:** Estimated model parameters.

	*β_d_*	*β_m_*	*γ_1_*	*p_I_*	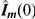
Animal Facility	-	-	0.27	0.66	-
Farm 1	8.22	1.06	0.43	0.68	1.93×10^−4^
Farm 2-F1	4.64	0.11	0.31	0.73	6.86×10^−3^
Farm 2-F2	3.88	0.25	0.35	0.51	4.83×10^−4^
Farm 2-F3	3.51	0.21	0.27	0.84	2.60×10^−3^
Farm 2-F4	4.89	0.14	0.33	0.58	1.41×10^−3^
**Mean** *	4.23	0.18	0.32	0.67	2.80×10^−3^
**S.D.** *	0.64	0.06	0.03	0.15	2.80×10^−3^
**Median** *	4.26	0.18	0.32	0.66	2.00×10^−3^

* Farm 1 is excluded for calculations of mean, S.D., and median.

#### Model-fit to farm data

Since the data available are limited (only 10 data points per farm), we simplify our full model for the sake of data fitting. Compared to the life expectancies of both ducks and mosquitoes, the duration of the data considered in this study is extremely short (only 10 days). Therefore, the natural death rates of both ducks and mosquitoes can be neglected for data fitting purposes, i.e. *μ_d_* = *μ_m_* = 0 d^−1^. Assuming that both ducks and mosquitoes are in equilibrium states before the infection begins, we also have *λ_d_* = *λ_m_* = 0 d^−1^.

Laboratory tests for BYDV-specific antibody using the ELISA method indicate that the BYDV-specific antibody level in recovered ducks is 3-fold higher than in control ducks [2]. This suggests that there might not be significant loss of immunity over the short period during which the data was collected. Since the exact duration of immunity in ducks infected by BYDV is unknown, we simply assume no loss of immunity, i.e. *η* = 0 d^−1^ over the 10-day data collection period. The total mortality rate of 5–15% observed during the study period in several duck farms in Southeast China may reflect the management conditions of the infected ducks [2] rather than representing actual disease mortality. Nonetheless, we assume 10% mortality over the 10-day data collection period, which approximately gives *δ* = 0.01 d^−1^.

Finally, a decline in feed uptake is one of the visible symptoms of this disease. Based on the average feed uptake rate of an infected flock [2], after infected ducks show symptoms and reduce their feed uptake, more than two weeks pass before normal feed uptake resumes. Therefore we conservatively assume that the time period for infected ducks to fully recover is three weeks, i.e. *γ_2_* = 1/21≈0.05 d^−1^. This implies that it is unlikely that any infected ducks will fully recover during the initial 10 days of the disease epidemic. We can therefore neglect egg production by recovered ducks for the purpose of data fitting. The egg production rate, 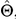 is then given by
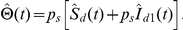
Due to the uncertainly in our estimate of *η* and *γ_2_*, we have also carried out a sensitivity analysis to observe how egg production is affected by varying these rates (see Figs. S1 and S2).

We assume that the virus is initially introduced by mosquitoes, and take 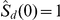 and 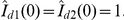 (Note that we also performed data fitting by allowing 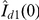 to be a free variable. In this case, the estimated 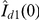 was extremely low (on the order of 10^−7^) and increasing the number of parameters by including 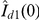 did not improve the model fit.) We estimate parameters *β_d_*, *β_m_*, *γ_1_*, *p_I_* and 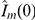 by minimizing the following sum of the squared residuals:
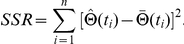
Here, 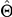 and 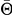 are egg production rates predicted by the model and those given by the data [2], respectively; *n* is the total number of data points. Note that the egg production rate for susceptible ducks, *p_s_*, is set to the value observed at time zero, which represents the egg production rate at the beginning of the epidemic. We solve system (3) using a 4^th^ order Runge-Kutta algorithm and carry out data-fitting using the nonlinear least squares regression method in Berkeley Madonna v8.3.18 [13].

Using the best fit parameters, we plot the egg production rate predicted by the model along with the data for each farm in Fig. 2. The predictions of our model (solid curve) agree well with the data (filled circles). The parameters estimated in this way are provided in Table 1. Despite the variable time courses of egg production seen among flocks (Fig. 2), we note a remarkable consistency in our estimates for *γ_1_* and *p_I_*, including those parameters independently estimated from experimental data at the animal facility. We discuss the discrepancy between estimates of the infection rates, *β_d_* and *β_m_*, for farm 1 and farm 2 in the next section.

## Results

### BYD disease characteristics

#### Transmission and progression

As revealed in the data (Fig. 2), transmission dynamics in farm 1 clearly differ from other flocks. While the data showed that all flocks were affected, resulting in significant drops in the egg production 10 days after infection, it is clear that the drop rate is quite different between farm 1 and farm 2; in farm 1 the egg production rate dropped from 90% to 10% in about 6 days, while in farm 2, it took about 10 days for the rate to drop from 60% to 10%. In agreement with this, our estimates of transmission rates, β_d_ and β_m_, for farm 1 are significantly higher than those for other flocks (t test, p = 0.0116 for β_d_ and p = 0.0011 for β_m_, Table 1). Notice that farm 1 consisted of a 35 week-old flock while others were 76 week-old flocks. This suggests that younger ducks are more vulnerable to BYD disease than older ducks, similar to many other diseases such as malaria. Thus the mean parameter estimates described below exclude parameters estimated for farm 1.

Our estimate of *γ_1_* = 0.32±0.03 d^−1^ (Table 1) implies that after infection by BYD virus, ducks remain capable of producing eggs for about 3 days, and during this stage, their production capacity is reduced to 67±15%. After this 3 day early infection stage, infected ducks become unable to produce eggs (or produce negligible eggs), consistent with the severe hemorrhage of the ovaries observed experimentally 3 days post-infection [2]. Although estimates of 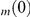 showed the greatest variation among farms, our results indicate that the average proportion of infected mosquitoes at the beginning of the data collection is 2.80±2.80×10^−3^ (Table 1). This shows that even low levels of infected mosquitoes can generate this epidemic, indicating that the virus is highly transmissible.

#### Long-term dynamics

**Figure 3:**
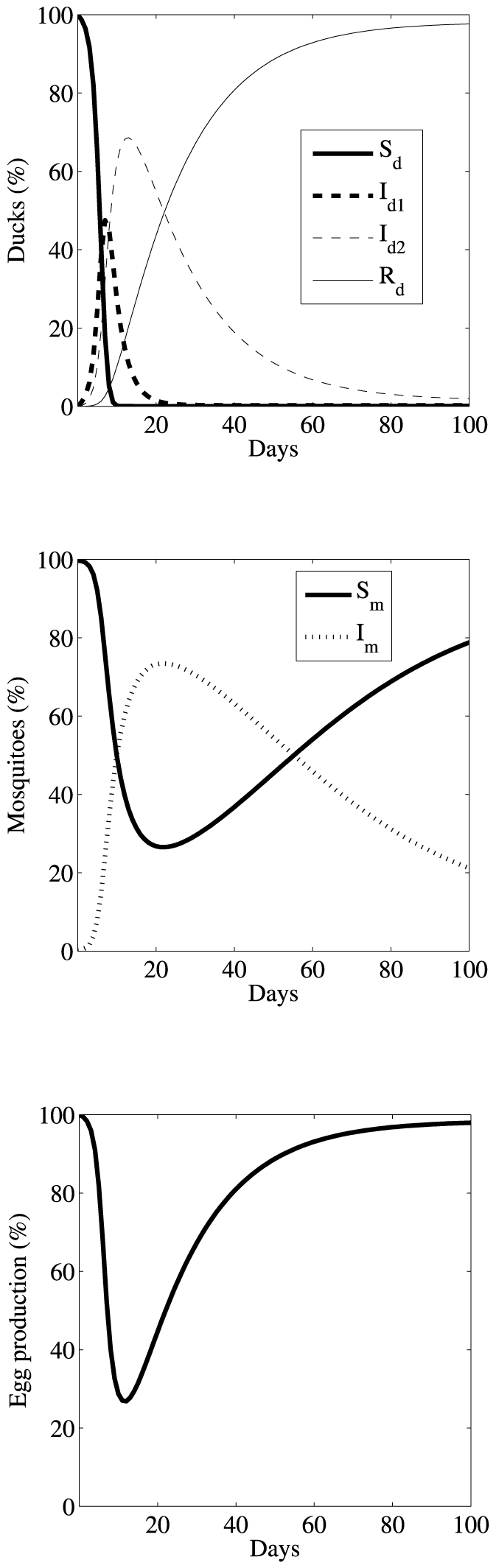
Long-term dynamics of (a) duck populations (b) mosquito populations and (c) egg production rate. Simulations are carried out using the mean values of the parameters in Table 1. Other parameters used are *η* = 0 d^−1^, *δ* = 0.01 d^−1^, 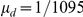 d^−1^ and 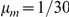 d^−1^.

Using the average values of the estimated parameters (Table 1) we simulate the time course of a typical outbreak, following both duck and mosquito populations and the egg production rate (Fig. 3). We again assume *η* = 0 d^−1^ (no loss of immunity) and *δ* = 0.01 d^−1^ as discussed above. We also perform a sensitivity analysis to observe how egg production is affected when the rate of immunity loss, *η*, varies (see Fig. S2). The life spans of ducks vary widely depending upon species. In this simulation, we take 3 years as the life span of ducks, i.e. *μ_d_* = 1/1095 d^−1^, and assume equilibrium pre-infection to calculate recruitment of ducks to the farm, i.e. *λ_d_* = *μ_d_S_d_*/*N_d_*. Since the life span of commercial ducks may be shorter than the natural life span due to depopulation, we also perform a sensitivity analysis to study the effects of the life span of ducks on the dynamics of the egg production rate (see Fig. S3). Moreover, we take a 1 month life span for mosquitoes, i.e. μ_m_ = 1/30 d^−1^ [14], and use λ_m_ = μ_m_S_m_/N_m_. Since the life span of mosquitoes varies widely, we also investigated the dynamics of the egg production rate assuming a mosquito life span of 1, 2, …, 6 week and find that the result is not affected by the choice of μ_m_ (data not shown as the curves are indistinguishable). Numerical integration of system (3–4) is carried out using the built-in function, ode23.m, in MATLAB (MathWorks, Inc.).

Based on the predictions of our model, BYD epidemiology within a newly infected flock can be classified in three successive phases: the acute phase (approximately 0–10 days), the transition phase (approximately 10 days – 2 months) and the recovering phase (past 2 months). The acute phase is associated with a dramatic reduction in the egg production rate, during which most susceptible ducks become infected resulting in a drastic reduction in the uninfected population. During the transition phase, most mosquitoes in the population carry the virus (Fig. 3). The beginning of the transition phase is characterized by the point at which egg production reaches its lowest rate; production recovers gradually as this phase progresses. The end of the transition phase occurs when susceptible mosquitoes again outnumber infected mosquitoes. By the time the recovery phase begins egg production returns to near normal levels (Fig. 3).

#### Reproductive number

Using the next generation matrix approach [15]–[17] (see appendix S1), we calculate the basic reproductive number, *R*_0_, which is defined as the average number of secondary infections generated by a single infected individual introduced into a completely susceptible population. *R*_0_ is regarded as a threshold value for an epidemic to die out (*R*_0_<1) or a disease outbreak to occur (*R*_0_>1) [7]. We obtain the following expression for *R*_0_ in our model:
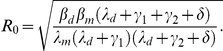
Using the estimated parameters (Table 1), we obtain *R*_0_ = 21 for BYD virus. This high value of *R*_0_ is consistent with the rapid and dramatic drop in egg production observed in BYDV outbreaks.

### Control strategies

Since successful vaccines have been widely used for many flaviviruses such as yellow fever virus and Japanese encephalitis virus, it has been speculated that a vaccine against BYD virus is feasible [2]. However, our prediction of 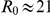 indicates that to reduce *R*_0_ below 1, about 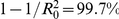 of ducks have to be protected by vaccination against BYD virus (see appendix S1). Based on this result, a vaccination strategy does not seem to be feasible for preventing BYD epidemics.

**Figure 4:**
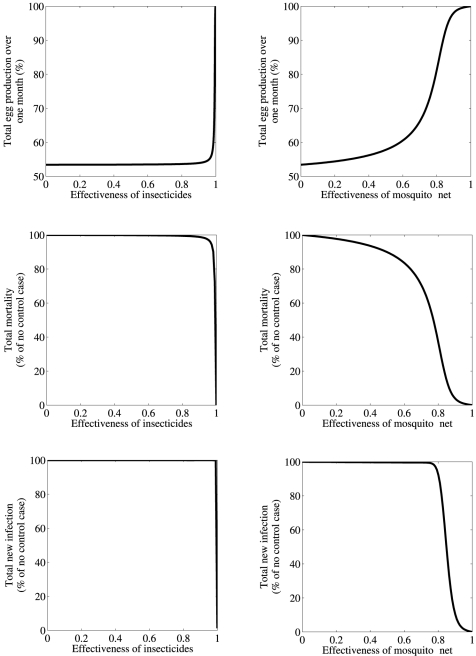
Effect of control strategies, (a) insecticide (b) mosquito-net, on the total egg production, the total mortality and the total new infection cases over a month (%). Simulations are carried out using the mean values of the parameters in Table 1. Other parameters used are *η* = 0 d^−1^, *δ* = 0.01 d^−1^, 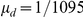 d^−1^ and 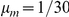 d^−1^. Here, egg production is expressed as the percentage of the total egg production in a month in the absence of BYDV. Mortality and new infection cases are expressed as the percentage of the total mortality and the total new infection cases in the absence of control, respectively.

Apart from vaccination, common practices for controlling mosquito-borne diseases are insecticides and mosquito-nets [18]. As insecticides reduce the mosquito population, the effects of this strategy on egg production can be studied by manipulating the mosquito population size in our model. Similarly, mosquito-nets reduce contacts between ducks and mosquitoes thereby decreasing the values of parameters *β_d_*, and *β_m_* in our model. Scaling the effectiveness of these strategies between 0 (no control) and 1 (perfect control), we study the impacts of these control strategies on the total monthly egg production (Fig. 4). Our results show that insecticides are less effective at preserving egg production and minimizing disease burden, as even a 99% reduction in the mosquito population is predicted to improve egg production, disease mortality and infection cases by less than 10%. This is in line with the fact that BYD virus can cause outbreaks even when a small proportion of mosquitoes are infected (Table 1). On the other hand, mosquito-nets are much more effective at preserving egg production, particularly if the effectiveness of the nets is higher than 60% (Fig. 4). In this case, both disease mortality and infection cases are also significantly reduced (Fig. 4). As expected a higher effectiveness maintains a higher egg production, and control strategies which reduce duck-vector contacts by 85% or more are predicted to maintain total egg production above 90%.

## Discussion

Baiyangdian (BYD) virus is a recently-isolated, and novel, avian pathogen which has devastating effects on duck egg production causing serious economic loss [2]. In addition to rapid spread among extremely large duck populations (about 4.4 millions ducks infected in three Chinese provinces alone), the zoonotic nature of this vector-borne disease may also put human health at risk [1], [2]. Understanding the epidemiology of this novel disease in duck populations may be useful to devise control strategies and prepare for the possibility of cross-species transmission.

In this study, we develop a mathematical model to study transmission dynamics of this novel virus among duck populations. The model provides an excellent fit to data recorded during natural outbreaks in 5 duck flocks and also to data obtained by experimental infection of ducks in an animal facility (Table 1). Epidemiological parameters estimated in this way are remarkably consistent including those for experimental ducks, providing validation of our model assumptions and parameters estimated. While there remains much uncertainty about this newly identified virus, our results offer some interesting findings. We show that BYD epidemics can be established even with a significantly low infected mosquito population (Table 1). This explains an ambiguity pointed out by Su et al. [2]: that the BYD virus infection in egg-laying ducks continued into autumn when mosquito activity is low in northern China.

We find a statistically significant difference in estimates of transmission rates between 35 week- and 76 week-old flocks (*t* test, p = 0.0116 for *β_d_* and p = 0.0011 for *β_m_*), indicating that younger ducks are primarily vulnerable to BYD virus. Using the best-fit transmission rates of BYD virus, we estimate the basic reproductive number to be 21, which indicates that the virus is extremely transmissible. As a flavivirus, BYD has the potential for cross-species and/or human-to-human transmission; if transmission rates in ducks reflect potential human transmission rates, BYD epidemics in humans could spread rapidly. In addition, as evidenced by epidemics such as the 1968 influenza epidemic [16] and H5N1 outbreaks in Africa, Asia, Europe and the Middle East [19], [20], zoonotic viruses which cross from birds to humans may be highly virulent in the human population. This result underscores the need for further detailed studies of BYD virus. Note that a newly emerged Tembusu virus strain, FX2010, which has nucleotide sequences for the E and NS5 genes similar to BYD virus, has already been found to be transmitted without mosquitoes in some species of ducks (e.g. shelduck) [21].

Our model predicts that ducks infected with BYD virus reduce their egg-producing capacity to 67% for 3 days post infection and after 3 days completely lose their ability to produce eggs. In general, even a healthy duck does not produce an egg every day, i.e. 100% egg production does not occur in uninfected farms. Since ducks infected with BYD virus are capable of 67% egg production for the first 3 days of infection, it may be difficult to detect BYD outbreak in its early stages, by simply monitoring egg production. This, combined with the rapid early transmission and high reproductive number, suggest that other mechanisms for early detection will be critical for effective BYD control.

Our model simulations indicate three successive phases in a typical BYD outbreak with acute, transition, and recovering phases of approximate duration of 0–10 days, 10 days – 2 months and past 2 months, respectively. Our sensitivity analysis shows that the duration of transition and recovering phases are mostly sensitive to the rate of immunity loss, *η*, while the duration of acute phase remains consistent over wide ranges of parameters. A rapid decline in egg production occurs during the acute phase, followed by a gradual recovery of egg production during the transition phase and finally, complete recovery. The acute stage dynamics are driven by rapid transmission, whereas the slow recovery phase is dominated by the relatively long duration (several weeks) of the *I_2_* stage of infection (Fig. 3). In such situations as rapid transmissions and slow recoveries, effective control strategies become particularly imperative. Using our model we also evaluate the effectiveness of two control strategies, insecticides and mosquito-nets. Our results reveal that a strategy of insecticide control is not effective, as BYD outbreaks may occur even with a significantly reduced initial proportion of infected mosquitoes (Table 1); only negligible improvement in egg production (less than 10%), disease mortality and total cases of infection can be achieved by reducing the mosquito population by more than 99%. We predict that mosquito-nets or similar strategies which reduce mosquito-duck contacts are a more efficient means of mitigating BYD disease and preserving egg production.

We acknowledge several limitations of this study. There are only limited studies to date on BYD virus and much information related to this novel virus is unknown or uncertain. While transmission of BYD virus among ducks is most likely via vectors such as mosquitoes as concluded from genomic sequence analyses [2], we cannot completely rule out the possibilities of other transmission routes such as direct duck-to-duck transmission [7] and/or environmental transmission [22]. As noted above, FX2010 virus, a newly identified Tembusu virus strain similar to BYD virus, was transmitted without mosquitoes in three naive shelducks in an experiment [21]. However, we found that fitting a model [7] with direct transmission only (without vectors) could not fit the available BYDV data well, as measured by Akike's information criterion (see Table S1). In addition, the parameters estimated in this case were not consistent with the parameters estimated using experimental data (see Table S1 and Fig. S4); this is particularly problematic for the direct transmission model since parameters estimated from the experimental data are independent of transmission route. These observations suggest that either small difference in the nucleotide sequences of the FX2010 virus provides the ability for FX2010 to be transmitted through different routes, or that transmission and/or progression patterns of these viruses vary with the host species. We note that most birds infected by FX2010 virus were shelducks, with no report of infection of Muscovy ducks [21], whereas BYD virus primarily infects Muscovy ducks, Peking ducks and domesticated mallards [2]. In addition, these virus infections also show pathological differences; for example, high levels of FX2010 virus were detected in the trachea [21] showing potential for direct transmission through respiration, whereas BYD virus in the trachea has not been reported [2]. Regarding the possibility of environmental transmission, we note that discharge of BYD virus by infected ducks has not been confirmed, and there is no knowledge of viral persistence outside hosts. Further experimental studies and modeling exercises are clearly needed to clarify these issues.

## Supporting Information

Appendix S1 Calculation of the reproductive number using the next generation matrix.
(PDF)

Figure S1 **Sensitivity of the total egg production over a month to changes in the recovery rate.**
(PDF)

Figure S2 **Sensitivity of the total egg production over a month to changes in the rate of immunity loss.**
(PDF)

Figure S3 **Sensitivity of the egg production dynamics to changes in the duck life span.**
(PDF)

Figure S4 **Egg production rate predicted by the model with direct transmission as compared to the model with mosquito-borne transmission.**
(PDF)

Table S1 Estimated parameters using model with direct transmission route.
(PDF)
