# Supplementary material for: Transmission Dynamics of the Recently-Identified BYD Virus Causing Duck Egg-Drop Syndrome
Source: PLoS One. 2012 Apr 18;7(4):e35161. doi: 10.1371/journal.pone.0035161 (PMC3329443; doi:10.1371/journal.pone.0035161)
Supplement: Appendix S1 — Calculation of the reproductive number using the next generation matrix. (PDF) [file pone.0035161.s001.pdf]

Appendix S1. Calculation of the reproductive number using the next generation matrix.

In this section, we derive R_0, the reproductive number, using the next generation matrix method [16-18]. It is easy to see that the scaled model system has exactly one disease-free equilibrium $E_0 = (1,0,0,1,0)$ and the equations for the infectious compartments of the linearized system of the scaled model at E_0 take the form:

$$\frac{d\hat{I}_{d1}}{dt} = -(\lambda_d + \gamma_1)\hat{I}_{d1} + \beta_d \hat{I}_m, \tag{9}$$

$$\frac{d\hat{I}_{d2}}{dt} = \gamma_1 \hat{I}_{d1} - (\lambda_d + \gamma_2 + \delta)\hat{I}_{d2}, \tag{10}$$

$$\frac{\hat{I}_m}{dt} = \beta_m\left(\hat{I}_{d1} + \hat{I}_{d2}\right) - \lambda_m \hat{I}_m. \tag{11}$$

We now introduce the following matrices:

$$F = \begin{pmatrix} 0 & 0 & \beta_d \\ 0 & 0 & 0 \\ \beta_m & \beta_m & 0 \end{pmatrix},$$

$$V = \begin{pmatrix} \lambda_d + \gamma_1 & 0 & 0 \\ -\gamma_1 & \lambda_d + \gamma_2 + \delta & 0 \\ 0 & 0 & \lambda_m \end{pmatrix}.$$

These expressions give

$$FV^{-1} = \begin{pmatrix} 0 & 0 & \frac{\beta_d}{\lambda_m} \\ 0 & 0 & 0 \\ \frac{\beta_m(\lambda_d + \gamma_1 + \gamma_2 + \delta)}{(\lambda_d + \gamma_1)(\lambda_d + \gamma_2 + \delta)} & \frac{\beta_m}{\lambda_d + \gamma_2 + \delta} & 0 \end{pmatrix}.$$

Then R_0 corresponds to the spectral radius of FV^{-1}:

$$R_0 = \rho(FV^{-1}) = \sqrt{\frac{\beta_d \beta_m(\lambda_d + \gamma_1 + \gamma_2 + \delta)}{\lambda_m(\lambda_d + \gamma_1)(\lambda_d + \gamma_2 + \delta)}}.$$

This expression is also obtained when derived from the original (unscaled) system of ODEs (Eq. 1). Note that in the scaled equation, S_0 = 1. If a proportion v of ducks is vaccinated at the beginning of the infection, the total susceptible population reduces to (1-v)S_0 assuming that the vaccine provides perfect protection against BYDV. In this case, the effective reproductive number under vaccination, R_0^v, is given by

$$R_0^v = \sqrt{\frac{\beta_d \beta_m (\lambda_d + \gamma_1 + \gamma_2 + \delta)}{\lambda_m (\lambda_d + \gamma_1)(\lambda_d + \gamma_2 + \delta)}(1 - v)} = \sqrt{1 - v}\, R_0.$$

Therefore the minimum vaccination proportion, v_{min}, required to bring the reproductive number below one (i.e. $R_0^v < 1$) is

$$v_{min} = 1 - \frac{1}{{R_0}^2}.$$
