# Supplementary material for: Transmission Dynamics of the Recently-Identified BYD Virus Causing Duck Egg-Drop Syndrome
Source: PLoS One. 2012 Apr 18;7(4):e35161. doi: 10.1371/journal.pone.0035161 (PMC3329443; doi:10.1371/journal.pone.0035161)
Supplement: Figure S2 — Sensitivity of the total egg production over a month to changes in the rate of immunity loss. (PDF) [file pone.0035161.s003.pdf]

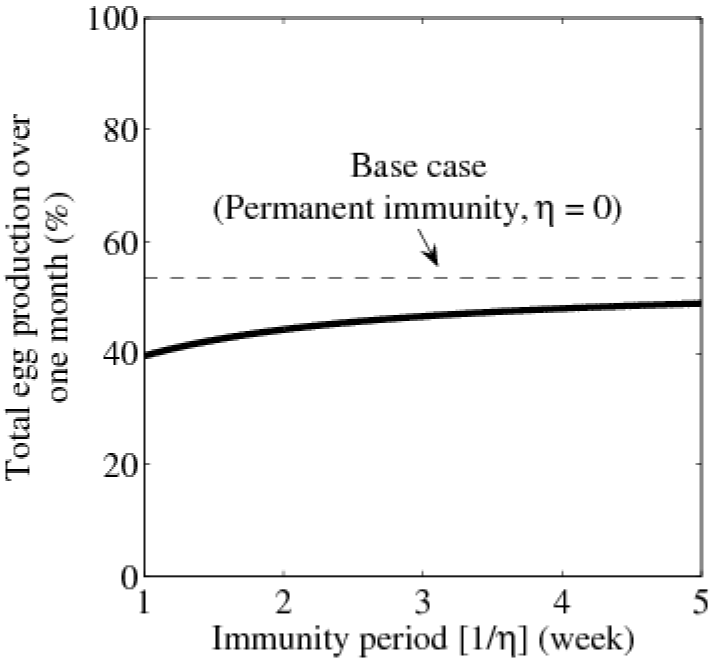

Figure S2. Sensitivity of the total egg production over a month (%) to changes in the rate of immunity loss, η. System (8-12) was integrated numerically, using the mean values of the parameters in Table 1. Other parameters used are $\gamma_2 = 1/21$ d^{-1}, $\delta = 0.01$ d^{-1}, $\mu_d = 1/1095$ d^{-1} and $\mu_m = 1/30$ d^{-1}. Here, egg production is expressed as the percentage of the total egg production in a month in the absence of BYDV.
