# Supplementary material for: Transmission Dynamics of the Recently-Identified BYD Virus Causing Duck Egg-Drop Syndrome
Source: PLoS One. 2012 Apr 18;7(4):e35161. doi: 10.1371/journal.pone.0035161 (PMC3329443; doi:10.1371/journal.pone.0035161)
Supplement: Figure S3 — Sensitivity of the egg production dynamics to changes in the duck life span. (PDF) [file pone.0035161.s004.pdf]

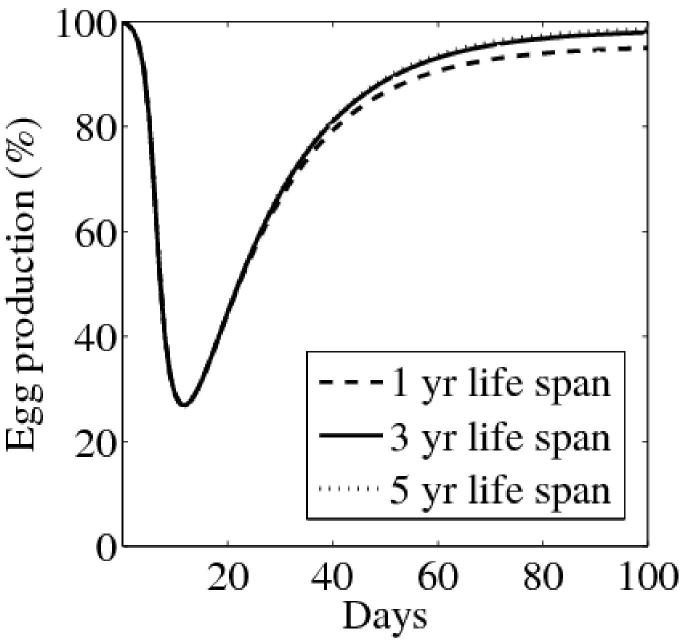

Figure S3. Sensitivity of the egg production dynamics to changes in the duck life span, $1/\mu_d$. System (8-12) was integrated numerically, using the mean values of the parameters in Table 1. Other parameters used are γ_2 = 1/21 d^{-1}, δ = 0.01 d^{-1}, η = 0 d^{-1} and $\mu_m = 1/30$ d^{-1}. Our results are clearly insensitive to the duck life span.
