# Supplementary material for: Transmission Dynamics of the Recently-Identified BYD Virus Causing Duck Egg-Drop Syndrome
Source: PLoS One. 2012 Apr 18;7(4):e35161. doi: 10.1371/journal.pone.0035161 (PMC3329443; doi:10.1371/journal.pone.0035161)
Supplement: Figure S4 — Egg production rate predicted by the model with direct transmission as compared to the model with mosquito-borne transmission. (PDF) [file pone.0035161.s005.pdf]

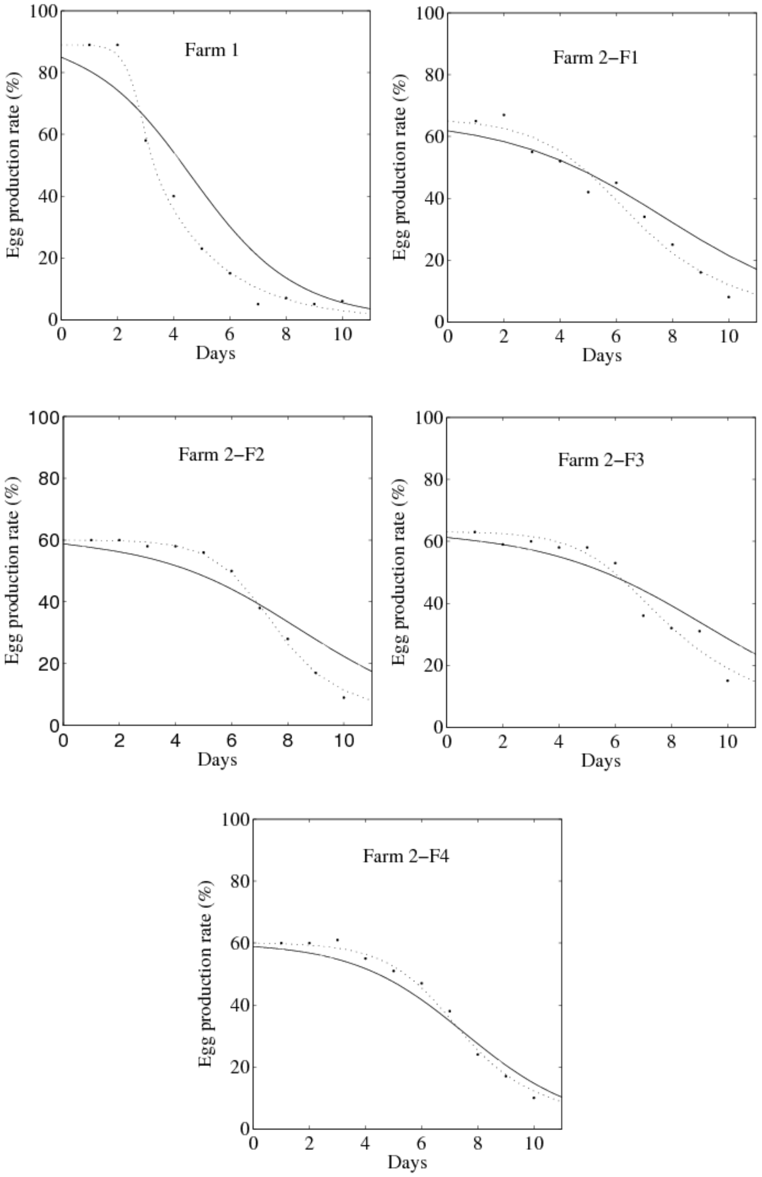

Figure S4. Egg production rate predicted by the model with direct transmission (solid line), the model with mosquito-borne transmission (dashed line) along with the data (dots). Egg production rate as expressed in % (y-axis) represents the percentage of ducks that produce eggs out of the total ducks in the flock on day *n*. See table S1 for estimated parameters with direct transmission and for a comparison of the goodness of fit.
