# Supplementary material for: Transmission Dynamics of the Recently-Identified BYD Virus Causing Duck Egg-Drop Syndrome
Source: PLoS One. 2012 Apr 18;7(4):e35161. doi: 10.1371/journal.pone.0035161 (PMC3329443; doi:10.1371/journal.pone.0035161)
Supplement: Table S1 — Estimated parameters using model with direct transmission route. (PDF) [file pone.0035161.s006.pdf]

Table S1. Estimated parameters using model with direct transmission route. The model system with direct transmission consists of 4 ODEs (excluding the mosquito population). The transmission term is modeled as $\beta(\hat{I}_{d1} + \hat{I}_{d2})S_d$. We fit this model to the same data. $AIC_c{}^D$ and $AIC_c{}^V$ represent Akaike's "information criterion" values for the model with direct transmission and the model with mosquito-borne transmission, respectively. Lower AIC_c values indicate better fits to the data. The model including vector-borne transmission always provides a superior fit.

	β	γ_1	p_I	$\hat{I}_{d1}(0)$	$\hat{I}_{d2}(0)$	$AIC_c{}^D$	$AIC_c{}^V$
Farm 1	8.22	1.06	0.43	0.68	1.93×10^{-4}	-2.52	-32.50
Farm 2-F1	4.64	0.11	0.31	0.73	6.86×10^{-3}	-13.10	-24.00
Farm 2-F2	3.88	0.25	0.35	0.51	4.83×10^{-4}	-13.12	-50.70
Farm 2-F3	3.51	0.21	0.27	0.84	2.60×10^{-3}	-15.43	-27.86
Farm 2-F4	4.89	0.14	0.33	0.58	1.41×10^{-3}	-24.26	-42.55
